# Effects of virtual reality v. biophilic environments on pain and distress in oncology patients: a case-crossover pilot study

**DOI:** 10.1038/s41598-021-99763-2

**Published:** 2021-10-12

**Authors:** L. Ashley Verzwyvelt, Ann McNamara, Xiaohui Xu, Renee Stubbins

**Affiliations:** 1 Houston Methodist Hospital, Cancer Center, 6445 Main St., 24th Floor, Houston, TX 77030 USA; 2 Department of Visualization, Texas A&M University, 3137 TAMU C108, College Station, TX 77840 USA; 3 Department of Epidemiology and Biostatistics, Texas A&M University, 212 Adriance Lab Road, College Station, TX 77843 USA; 4 Houston Methodist Hospital, Cancer Center Infusion, 6445 Main St., 21st Floor, Houston, TX 77030 USA

**Keywords:** Quality of life, Cancer, Oncology, Pain, Psychophysics

## Abstract

This pilot study aimed to determine if a biophilic Green Therapy or Virtual Reality environment can decrease an oncology patient’s pain and distress while receiving chemotherapy. A case-crossover pilot study was conducted in a comprehensive cancer infusion center. 33 participants with breast, gynecologic, gastrointestinal, pancreatic and prostate cancers were all included in three rooms in a random order at different cycles: control room, Green Therapy room, and Virtual Reality room to receive chemotherapy, respectively. Participants’ pain, distress, heart rate, blood pressure, and saliva cortisol were measured before and after infusion in each room. No statistical significance differences were shown in the changes of heart rate, systolic, or diastolic blood pressure, saliva cortisol, pain, or distress before and after infusion between the control, Green Therapy, and Virtual Reality rooms. However, more patients reported the experience as “fun” and “enjoyable” when they were in Green Therapy or Virtual reality room as compared to in the control room. Additionally, since participating in the study, 14 patients reported spending at least 30 min or more outside in nature. In this study, we found that patients’ heart rate, blood pressure, and self-reported distress levels were reduced after each biophilic intervention although results are not statistically significant. The study also suggested that biophilic interventions are safe and feasible and may complement the standard of care for oncology patients.

## Introduction

Oncology patients undergoing chemotherapy have a multitude of stressors impacting them during their infusions. These may include travel to appointments, side effects from treatments, anxiety, pain, and distress. Chemotherapy infusions can last up to eight hours and can be an emotionally daunting experience. Because these patients are already dealing with the chronic conditions resulting from their cancer, they are at an increased risk to experience distress compared to other patients. Moreover, 9 out of 10 oncology patients are on narcotics to control their pain; and 50–70% state that their pain is uncontrolled, and fear pain over death^1^. However, patients and providers are recognizing the value of safe complementary and alternative medicine (CAM) to control both distress and pain; especially since the opioid epidemic has made obtaining pain medication a difficult task^2^. Additionally, oncology patients often have unmet psychological concerns that can be difficult to assess^3^; however, several cancer centers use a distress screening tool as a first step. The distress screening tool is given to all new chemotherapy patients. Although we are proactively addressing both pain and distress in our oncology patients, we utilized the opportunity to use nature to manage both of these chronic conditions using safe CAM, specifically utilizing a biophilic Virtual Reality (VR) and Green Therapy (GT).

Virtual Reality allows users to experience computer-generated content and interact with it as they would in a real physical environment^4^. While VR has been around for a few decades now, recent advances have seen a resurgence in interest surrounding VR applications. The advantage of VR is that it enables users to interact with virtual environments in a manner that may not be possible in the real world. VR can create a realistic sense of “presence” within the computer-generated environment. The user is fully immersed in a three-dimensional (3D) virtual world that can be modeled to evoke a sense of place, including natural features and systems (e.g., forests, oceans, lakes, mountains). The use of VR as a treatment is growing. Research has shown improvements through VR intervention in treatment for conditions such as depression^5–7^, and sleep^8^. In a pilot study at St. Georges Hospital^9^, patients unanimously agreed using VR application to view calming landscapes during an operation improved their hospital experiences. 80% reported feeling less pain, while 94% said they felt more relaxed. Patients suffering from other conditions also reported lesser pain levels when VR was used to distract them. Virtual reality also allows the user to bring nature indoors. For those patients with limited mobility, this provides an alternative means to potentially benefit from interactions with nature.

Positive associations between nature and the health of humans continue to be shown through growing research. Epidemiological studies have demonstrated exposure to nature (e.g., green spaces), can effectively reduce stress, improve mental health, and overall patient outcomes, possibly due to the human innate attraction to nature and natural processes (i.e., biophilia)^10,11^. Recent studies have concluded that a biophilic/nature imagery can significantly improve patient satisfaction^12^. Additionally, research has shown that evidence-based healthcare design not only improves patient outcomes, but also is effective with ensuring patient safety and hospital staff outcomes^13^. There is a growing interest in reinventing hospital design to foster the human and nature relationship and it’s role in healing the body and mind^14^. Green Therapy (GT)/gardening can improve the health behaviors of cancer survivors, and the availability of green space can benefit cancer patients’ emotional and spiritual wellbeing^15,16^. However, it is unknown if the use of biophilic VR or GT can impact oncology patients’ distress and pain levels while actively receiving chemotherapy. Limited research has been done on using Green Therapy as a complementary therapy on patients actively receiving chemotherapy. By presenting a biophilic environment in an immersive VR system or in-person (GT), we would determine whether they positively impact oncology patients’ outcomes, specifically decreasing pain and distress while actively receiving chemotherapy.

## Methods and tools

### Study design and participants

Our study consisted of a case-crossover design to include 36 first-cycle oncology patients receiving intravenous chemotherapy. The study was overseen and approved by the Institutional review boards of Houston Methodist Hospital and Texas A&M University, and all experiments performed in accordance with relevant guidelines and regulations. Each participant was diagnosed from stages 0 to IV, with solid tumor origin including breast, cervical, endometrial, ovarian, colorectal, GE junction, pancreatic, and prostate cancers. Participants were identified, recruited, and received chemotherapy at Houston Methodist Cancer Infusion Center every two or three weeks for a total of three cycles. Exclusion criteria included hematology patients and/or patients already receiving chemotherapy. Participants were given an explanation of the study as well as study objectives. Consent was obtained at the participants’ first infusion appointment. Each of the participants received chemotherapy once in a private control room, GT room, and a VR experience room, respectively. The order of room occurrences was randomly assigned. With each occurrence, participants’ pain, distress, heart rate, blood pressure, and saliva cortisol were assessed pre and post-infusion.

### Data collection tools

#### Pain scale

An adaptive faces and numerical pain scale were used to assess each participant’s pain before and after each chemotherapy treatment in each of the assigned therapy rooms. The scale ranges from 0 (no pain) to 10 (excruciating/unbearable). The pain scale metric is a standard pain assessment tool and is used throughout all units and Houston Methodist Hospital locations. Patients’ self-reported numerical pain rating was logged at each session before and after the chemotherapy infusion.

#### Distress screening tool

The Houston Methodist Cancer Service’s Distress Screening Assessment Tool was used to assess emotional, social, physical, and spiritual distress for all new patients. This tool has a Distress Scale with scores from 0 (no stress) to 10 (extreme stress) and level 5 denoting a moderate level of stress. Patients’ self-reported distress level was logged at each session before and after the chemotherapy infusion.

#### Additional stress measurement tools

GE Dinamap Procare 400 was used to assess each participant’s blood pressure and heart rate before and after each infusion treatment. Vital signs were obtained in each of the assigned therapy rooms, and results were recorded at each visit.

#### Saliva cortisol testing

Saliva cortisol was used to assess each participant’s stress level before and after their infusion in each of the assigned therapy rooms. Specimen collection times were between 8 and 10 am for pre-infusion collection, and between 12 and 2 pm for post-infusion collection for optimal results. Lab specimens were then sent for analysis at the Houston Methodist Hospital Core lab using Roche Diagnostics technology. Roche Diagnostics saliva cortisol laboratory testing has a reference range of =  < 0.69, with the sensitivity ranges from 0.036–63.4 mcg/dL. Limit of Blank (lowest range detected) has been reported at < 0.018 mcg/dL. Values above max range though will not be detected. The lab literature provided stated their 326 tested sample concentrations were between 0.05 and 1.83 mcg/dL. The study that did their testing levels at 6 am, 9 am, and 9 pm established a classification from pooled mean cortisol of 0.25 mcg/dL or less as "low stress" group and anything above was classified as "high stress."

### Intervention

#### Control room

The control room is a standard treatment room that has no windows. The room is equipped with a standard infusion chair, vital signs monitor, and television. While in the control room, patients received standard of care during their chemotherapy infusion. Vital signs, saliva cortisol collection, pain, and distress assessments were conducted pre and post-infusion. No study intervention was conducted in this room.

#### VR room

**Figure 1:**
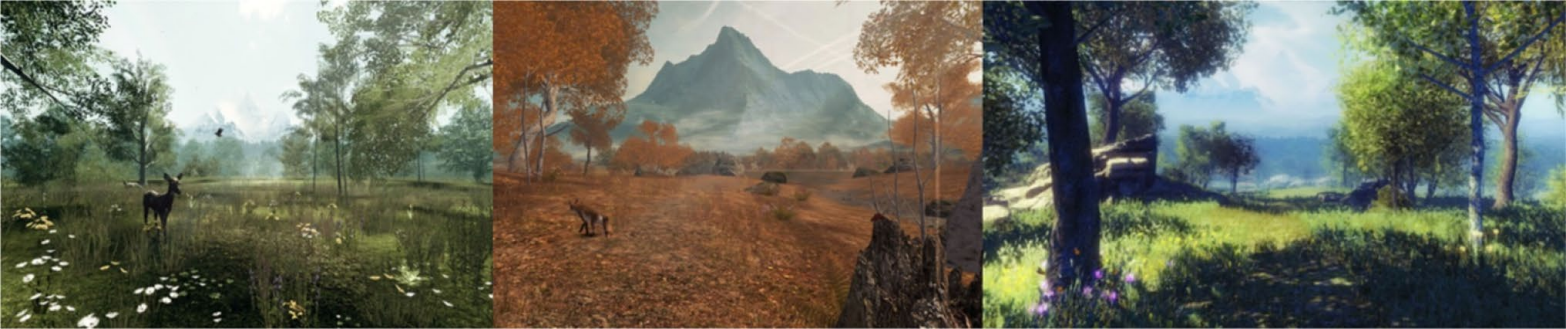
Nature treks environments.

The VR intervention was comprised of the Nature Treks software^17^, deployed on an Oculus Quest Head Mounted Display (HMD). The VR HMD is a stand-alone device with built-in tracking and headphones (to allow audio). The Oculus Quest has head tracking and a separate hand-controller to allow them to interact with the virtual environment. Patients were instructed to wear the headset for a minimum of 5-to-15-min intervals as tolerated throughout their infusion. Patients were able to choose between 9 different interactive natural environments. Patients can *“explore tropical beaches, underwater oceans and even take to the stars. Discover over 60 different animals. Command the weather, take control of the night or create and shape your own world.”* Total time using the headset was recorded in addition to vital signs, saliva cortisol collection, pain, and distress measurements (Fig. 1).

#### Green therapy room

**Figure 2:**
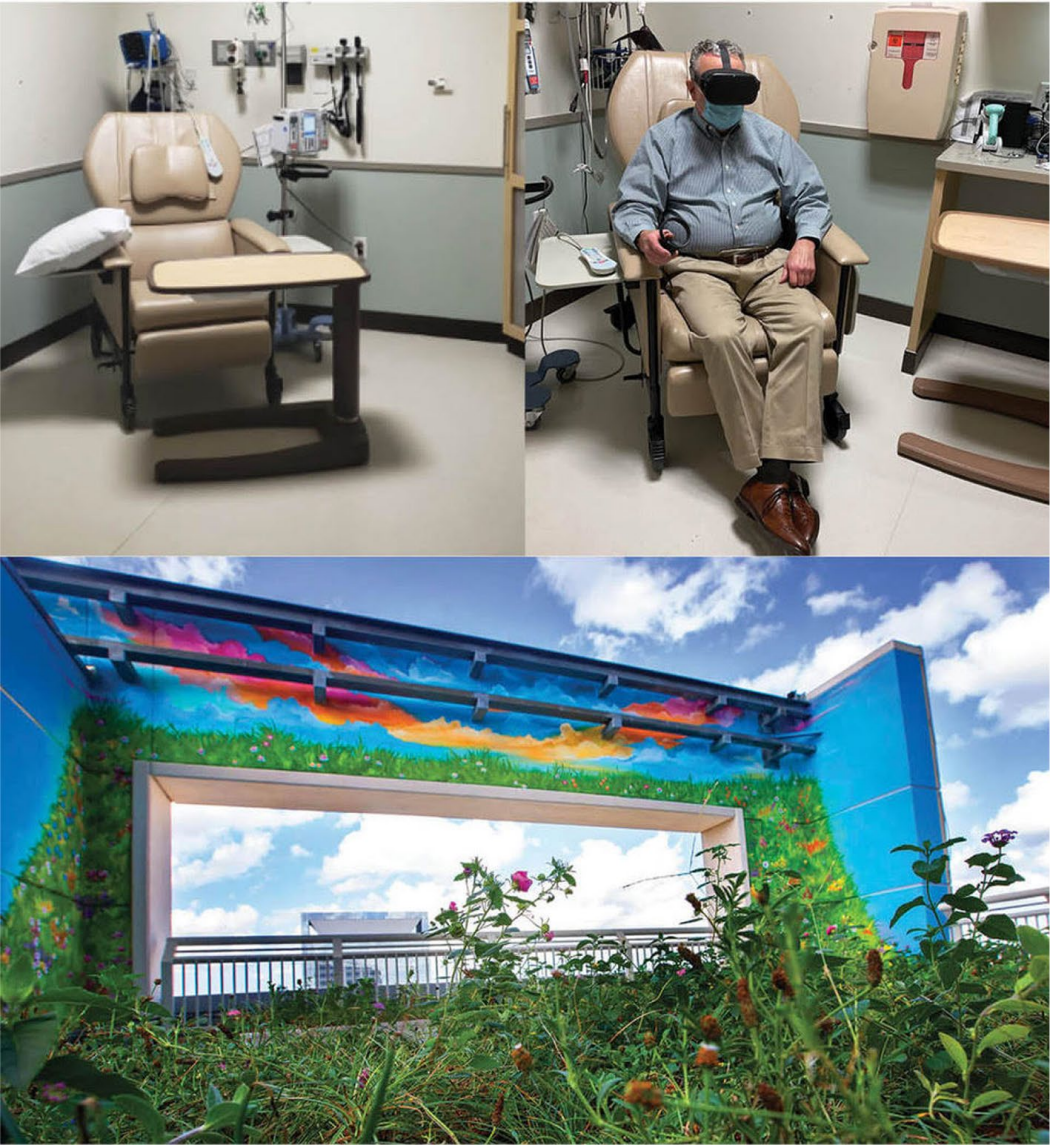
Study rooms and equipment.

The GT intervention placed the patient in a window-lined room. Patients placed in the Green Therapy room during their chemotherapy infusion were positioned to sit facing the large wall of windows overlooking a rooftop garden and picturesque mural, see Fig. 2. Vital signs, saliva cortisol collection, pain, and distress assessments were conducted pre and post-infusion.

### Statistical analysis

Descriptive analysis of demographic variables (age, gender, cancer stage, and cancer type) and outcome variables (heart rate, systolic blood pressure (BP), diastolic blood pressure (BP), saliva cortisol, pain, and distress levels) was tabulated with mean and standard deviations for continuous variables and total numbers and percentages for categorical variables. To test whether the distribution of the differences in outcome variables are Gaussian, the Shapiro–Wilk test was conducted. For normally distributed variables, ANOVA followed by posthoc Tukey HSD test was implemented to compare the equality of the differences in means and pairwise significant differences pre and post-chemotherapy across all treatments. For nonparametric distributed variables, Kruskal–Wallis followed by posthoc Dunn's test with Benjamin-Hochberg adjusted p-values were implemented to compare the equality of the differences in medians and pairwise significant differences pre and post-chemotherapy across all treatments.

### Ethics approval and consent to participate

The Institutional review boards of Houston Methodist Hospital and Texas A&M University approved the study protocol. Informed written consent was obtained by each participant.

## Results

### Patient demographics and cancer type/stage.

Study recruitment totaled 42 patients which allowed for a 15% screen failure rate. We had a power analysis done for 36 patients to allow for this screen failure. From the 42 recruited patients, nine withdrew and/or did not complete the study. Reasons for withdrawal included treatment plan change, prolonged hospitalizations, change of insurance, time conflict, and hesitation of VR headset use.

The results of our study are based on the 33 patients that completed the trial. We had a wide range of inclusion criteria that is reflected in the diverse characteristics of our patients. These are summarized in Table 1. The average age of our study population was 59 years old, with our youngest patient being a 26-year-old female and our oldest patient was an 84-year-old female. Most of our patients were female (n = 25), which is also reflective of our most common type of cancer in the study, breast cancer (n = 13). Additionally, almost half, 42.4% of our patients would be classified as advanced cancer patients, stage III-IV.

**Table 1:** Patient demographics and cancer type/stage.

Characteristics	Description	Avg ± SD (range)	Cancer Type	N(%)
Age		59.03 ± 13.2 (26–84)	Breast	13(40%)
			Cervical	1(3.0%)
Sex	Male	8(24.2%)	Colorectal	6(18.2%)
	Female	25(75.8%)	Endometrial	3(9.1%)
			Ovarian	1(3%)
Stage	0/I	6(18.2%)	GE Junction	1(3%)
	II	7 (21.2%)	Pancreatic	7(21.2%)
	III/IV	14 (42.4%)	Prostate	1(3.%)
	Unknown	6(18.2%)		

### Primary outcomes

#### Biological results in the control, green therapy, and virtual reality rooms before and after chemotherapy infusion

We did not find any statistical significance in heart rate, systolic, or diastolic blood pressure, saliva cortisol, pain, or distress between the control, Green Therapy, and Virtual Reality rooms. Additionally, there was no significant difference between each of the room’s biological measurements before and after chemotherapy infusion. However, it should be noted that the heart rates did decrease in both the Green Therapy and Virtual Reality rooms after chemotherapy infusion. Our biological measurements had a considerable amount of variability that could be partially explained by the various stages and types of cancer that were in our study. These results are shown in Table 2.

**Table 2:** Biological Results in the Control, Green Therapy and Virtual Reality rooms before and after chemotherapy infusion: Mean (s.d.).

	Control		Green therapy		Virtual reality	
	Pre-chemo	Post-chemo	Pre-chemo	Post-chemo	Pre-chemo	Post-chemo
Heart rate	78.2 (± 18.7)	77.6 (± 11.6)	81.7 (± 15.7)	77.9 (± 14.3)	79.6 (± 14)	77.7 (± 11.5)
Systolic blood pressure	128.2 (± 21.6)	124.7 (± 22.8)	130.9 (± 19.2)	128.3 (± 20)	131 (± 25.8)	122.5 (± 17.3)
Diastolic blood pressure	69.5 (± 7.5)	68.5 (± 8.4)	70.3 (± 8.0)	69.4 (± 7.7)	69.5 (± 8.9)	67.2 (± 7.5)
Saliva cortisol	0.3 (± 0.2)	0.3 (± 0.5)	0.4 (± 0.4)	0.3 (± 0.2)	0.4 (± 0.5)	0.4 (± 0.6*)
Pain	0.4 (± 1.6)	0.1 (± 0.5)	0.5 (± 1.9)	0.3 (± 1.2)	0.4 (± 1.2)	0.7 (± 1.9)
Distress	2.2 (± 2.1)	1.4 (± 1.7)	2.8 (± 3.1)	1.5 (± 2.0)	2.2 (± 2.7)	1.7 (± 2.6)

#### Patient usage of virtual reality

When the patient was assigned to the Virtual Reality (VR) room, they would be placed in a private room with no windows and would have access to an Oculus Quest headset. The patient would be oriented to the headset by the research coordinator and would be given a tutorial on how to interact with the Nature Treks application. The patients were encouraged to use the Oculus headset at their leisure. Our results are summarized in Table 3. The average usage of time was 53 min and the median time usage was 40 min. The maximum time usage was 150 min and the minimum usage was 10 min.

**Table 3:** Patient usage of virtual reality.

	Minutes
Mean	53.3
Median	40
Min	10
Max	150

### Secondary outcomes

#### Post-study questionnaire

After completing the study, patients were asked to complete a post-study questionnaire. Thirty-two patients completed the questionnaire and one patient did not due to missed follow up. Our post-study questionnaire included three questions and an open comment section. Approximately, 44% of patients reported that they were spending more time outside since starting our study. Specifically, 71.4% reported spending at least 60 min outside. Additionally, over 90% of patients reported that they are interested in the effects of nature on their health. These results are summarized in Table 4.

**Table 4:** Post-study questionnaire.

Post-study question	Minutes	N (%)
Since starting our study, are you spending more time outside?	Yes No	14/32 (43.75%) 18/32 (56.25%)
If yes, how much time would you say you are spending outside?	> 30 min	4/14 (28.6%)
	> 60 min	5/14 (35.7%)
	> 90 min	5/14 (35.7%)
Since starting our study, are you more interested in the effects of nature on your health?	Yes No	29/32 (90.63%) 3/32 (9.37a5)

In Table 5, we have included some of the comments patients shared with us regarding their experience while participating in our study.

**Table 5:** Patient Comments.

“So glad I participated in this study? It really makes it pleasant to come to chemo.”		“I want to thank you for your time and effort with this study, which was very interesting.”
	"The nature show I saw today was wonderful. I love and appreciate it and others. Thanks."	
“This made me appreciate the beauty of nature even more and looking forward to spending more time in my garden or going to parks.”		“Garden room was by far my favorite room, having warm sunshine was just what the doctor ordered.”
	“I love the garden room, it was so beautiful.”	
“I love the virtual reality goggles.”		“This greatly helped!”

## Discussion

The establishment of this study was to investigate the effects of pain and distress in a biophilic GT or VR environment on patients receiving chemotherapy. Although no significant changes were found in primary outcomes from the control room versus the biophilic rooms, secondary outcomes did indicate most participants were interested in learning about the effects of nature on health. The variability in age, cancer type, and cancer staging that is reflected in Table 1 was our intent to create an inclusive study. The most common type of cancer type in the study was breast cancer (40%), followed by pancreatic (21.2%), colorectal (18.2%), and endometrial (9.1%). Cervical, ovarian, GE junction, and prostate followed respectively at 1% of the participants. Almost half of the patients (42.4%) of the patients were stages III or IV, followed by stage II (21.2%), and stages 0 or 1, and unknown stage at 18.2%.

### Biological results

A small reduction in heart rate and blood pressure results occurred in each of the three rooms from pre to post-infusion. Saliva cortisol levels in each of the three rooms showed little overall change. Although few patients did experience reductions in pain and distress from pre-chemotherapy to post-chemotherapy infusions, the results were not statistically significant. It should also be noted that two participants experienced acute hypersensitivity reactions during their infusions in the VR and GT rooms. These participant variabilities may also have contributed to the lack of statistical significance in the results.

### Virtual reality use

Engaging, interactive, and automated virtual reality (VR) treatments might help the pain management needs of individuals. The application is intended to put the user into a peaceful, calming world to encourage relaxation. We tested the efficacy of a VR intervention for patients undergoing chemotherapy treatment. The study participants ranged in ages from 26 to 84. Despite the large gap and little no VR experience in the older patients, no true learning deficit was apparent. With a brief orientation and simple instructions, patients were able to navigate through their chosen environments and select other options without issue. With a maximum of 150 min, a median of 40 min, and mean of 53.3 min of VR usage, it is evident that the patients had a positive experience.

### Patient feedback

Post-study, an overwhelming majority of patients (90.63%) stated they were interested in learning how nature could impact their health. Almost half of the patients were spending more time outdoors since starting the study, with more than half spending greater than an hour to an hour and a half more as compared to pre-study. Patients of all ages were in awe of the variety of nature-themed environments offered in their VR intervention, and the ability to hear and interact within those environments. Patients commented on having a renewed appreciation for the beauty of nature and no longer dreaded coming to chemotherapy appointments. Although most of our feedback was positive, some patients did report that they would have found direct interaction with the outdoor garden more beneficial. Therefore, in our future studies and endeavors, we plan to expand our research to include mobile patient gardening so patients receiving chemotherapy will be able to directly interact with nature with more of their senses, i.e. site, smell, touch.

### Study strengths

A major strength of our study was the use of a landscape architect to meticulously design and layout a rooftop garden that provided optimum viewing from multiple GT rooms. Additionally, our VR expert provided thorough training for the headset to ensure patients were able to easily navigate through the nature program. From the results of the patient questionnaire, a surprising number of “older” patients outside of the “target audience” for VR reported the experience as “fun” and “enjoyable.” Additionally, the positive reports from patients who enjoyed viewing the garden, and the information obtained through this study will enable us to provide additional CAM therapy for our future projects and to consider offering these complementary therapies as part of our standard care, similar to music and art therapy already used in several cancer centers.

### Study limitations

Our study had a few limitations. These included a relatively small sample size with a large variety of cancer types and stages. Given the large number of breast cancer patients in the study, our male patients were underrepresented. This consideration should be examined for future studies. Pre-medications given to patients before starting chemotherapy can have sedative effects. Those patients sensitive to anticholinergic medications limited the amount of VR use and GT viewing due to drowsiness. Additionally, dry mouth side effects inhibited a couple of saliva cortisol collections/results.

## Conclusion

Our moderately small sample size of patients with a wide variety of ages, cancer types, and stages may have reflected on the lack of differences and power in our results. For our futures studies, we have refined our inclusion criteria (i.e. cancer type and stage(s)) to potentially strengthen the study significance.

Overall, we found that oncology patients valued their experiences with nature and benefitted from the opportunities to connect with it during and after treatment. The engagement of nature throughout the study eased some of the burden experienced by patients during treatment and encouraged them to further explore its benefits.

In conclusion, although significant reductions in patients’ pain and distress primary outcomes were not achieved, the biophilic environment rooms are a feasible and safe complementary therapy for oncology patients.
